# The impact of official development assistance on the economic growth and carbon dioxide mitigation for the recipient countries

**DOI:** 10.1007/s11356-020-10138-y

**Published:** 2020-07-22

**Authors:** Sue Kyoung Lee, Gayoung Choi, Eunmi Lee, Taeyoung Jin

**Affiliations:** Division of Climate Technology Cooperation, Green Technology Center, 173 Toegye-ro, Jung-gu, Seoul, 04554 Korea

**Keywords:** Official Development Assistance, Recipient countries, Economic growth, CO_2_ emissions, Environmental Kuznets curve

## Abstract

The purpose of this study is to investigate the relationship between official development assistance (ODA) on CO_2_ emissions based on both direct and indirect frameworks, using the annual panel data of 30 recipient countries of Korea from 1993 to 2017. It employs a modified impact, population, affluence, and technology (IPAT) model and a simultaneous equation framework for the direct model and indirect model, respectively. The empirical results suggest that ODA has both a direct and an indirect mitigation impact in the recipient countries. Compared to the direct impact, a small indirect mitigation impact of ODA on CO_2_ emissions is derived. However, the estimation results of the environmental Kuznets curve (EKC) equation imply that economic growth has the potential of mitigating the environmental degradation when the economic development in recipient countries of Korea reaches a certain level. Therefore, the bilateral cooperation, through ODA and the supportive policy, should make an effort to promote economic development and mitigation of environmental degradation in developing countries.

## Introduction

Since the Paris Agreement was adopted in December 2015, at the 21st Conference of Parties to the United Nations Framework Convention on Climate Change (UNFCCC), the climate change regime has shifted from the actions within the Kyoto Protocol to the new era. Although past negotiations at the UNFCCC have failed to draw binding forces, experts prospect that the Paris Agreement will be a turning point in global efforts to mitigate climate change (Kinley 2016). Literally, this is the first global accord containing policy obligations for all countries that have ratified it (177 countries out of 193 official countries). The Paris Agreement covers mitigation of greenhouse gas (GHG) emission, adaptation on climate change, and global cooperation via financing (Işik et al. 2018). It entered into force under the UNFCCC to find solutions for climate change and accelerate sustainable development (Dimitrov 2016).

As mentioned in the Paris Agreement, the main aim of the agreement is “to strengthen the global response to the threat of climate change by keeping a global temperature rise this century well below 2 °C above pre-industrial levels and to pursue efforts to limit the temperature increase even further to 1.5 °C.”^1^ In other words, since global efforts to prevent climate change due to global warming are required, curbing the temperature change in the atmosphere, which is closely related to greenhouse gases, is of utmost importance GHGs emission, which mainly consist of carbon dioxide (CO_2_), has been identified as a driving factor of climate change (Kang 2015), and its rapid increase triggers climate change, with its negative impacts being experienced within the natural ecosystem (IPCC 2007).

Given that CO_2_ mitigation is one of the most urgent assignments in the fight against climate change, global efforts to mitigate CO_2_ emissions are slowly taking root. As one of the modalities for mitigating climate change under the Kyoto Protocol, a carbon offset scheme, such as the Clean Development mechanism and Joint Implementation, has been put in place and its unique market, called the emission trade scheme, has been vitalized. Other than these efforts, global cooperation is also part of the efforts aimed at enhancing climate change mitigation. Official development assistance (ODA), which is the financial aid aimed at promoting the economic development and welfare of developing countries, should play a crucial role in the fight against climate change. Developing countries are considered to be more vulnerable to climate change than developed countries because they are relatively slow in taking appropriate actions to mitigate climate change and they highly depend on industries, which are the main triggers of climate change (Kang 2009). Michaelowa and Michaelowa (2007) argue that the main function of ODA is changing due to the emerging climate change issue. Organization for Economic Co-operation and Development (OECD 2009) also suggested integrating climate change adaptation into development cooperation. However, there is no current study covering the effectiveness of ODA in mitigating climate change.

Overseas capital flow consists of foreign direct investment (FDI), ODA, and migrant remittances (Driffield and Jones 2013). ODA is closely related to the economy of developing countries because foreign capital can be absorbed to developing countries through ODA. Related literature has provided a substantial amount of evidence describing the role of ODA in the economic growth of developing countries (Siraj 2012). In general, the foreign capital inflow through ODA projects promotes the economic growth of a recipient country in instances where the said country has appropriate policies in place (Burnside and Dollar 2000). On the contrary, one of the pioneers in the field, Griffin and Enos (1970), expressed concerns that foreign aid, such as ODA, can hinder economic growth. Momita et al. (2019) suggest that before debating on the effectiveness of ODA, researchers should take the heterogeneity of ODA into account because it might be the factor that influences the impact of ODA on economic growth in a recipient country.

According to Awan (2013), maintaining environmental quality should be pursued to the global goal, sustainable development. All human activities involving economic development affect environment one way another. Accordingly, to increase the sustainability, environmental regulation for any business, especially in the global context is essential (Gemmell and Scott 2013). It directly connects to the fact that global aid such as ODA should consider the environmental regulation and environmental impact of business and project.

The purpose of this study is to investigate and measure the impact of ODA on CO_2_ emissions. Since we adopt ODA inflow data, research subject is accordingly the recipient countries. In the context described above, ODA indirectly impacts CO_2_ emissions given that the relationship between economic growth and environmental degradation has been extensively discussed in a lot of literature. This study adopts CO_2_ emissions for environmental degradation indicators based on the fact that CO_2_ is at the center of mitigating GHG emissions. To investigate the role of ODA in carbon mitigation, we construct two equation frameworks: first, we measure the direct impact of ODA on CO_2_ emissions under the consideration of economic growth and population effect, and second, we estimate the simultaneous equation to establish the indirect pathway from ODA to CO_2_ emissions via economic growth.

For the estimating method, we adopt panel data analysis. Generally, panel analysis is considered to be more powerful than time-series analysis because of its affluent dataset (Harris and Tzavalis 1999). In econometric analysis, the data without the outlier directly connect to the informative power (Jin and Kim 2018a). Moreover, in the analysis for developing countries, the importance of using much data is emphasized because data for developing countries are not well constructed. This is the first study to quantify the direct and indirect impact of ODA on CO_2_ emissions, and its findings, therefore, contribute to the existing literature.

This study is organized as follows. The “Literature survey” section presents the existing literature related to ODA, economic growth, and carbon emissions. The “Data and the empirical model and methods” section proposes the data we collect, the estimation model, and methods. The “Empirical results” and “Conclusion” sections outline the empirical results and conclusion, respectively.

## Literature survey

### The impact of ODA on the economic growth of recipient countries

A lot of research covering the relationship between ODA and economic growth in recipient countries has been conducted. ODA recipient countries, which are territories eligible to receive financial aid, are classified as such by the World Bank and the United Nations, and the OECD publishes a Development Assistance Committee (DAC) list that shows all the countries and territories eligible to receive ODA. ODA recipients consist of upper-medium-income countries, lower-medium-income countries, and least-developed countries (LDCs), which are all representatives of developing countries. Most researches employ a panel data analysis that is capable of conducting multi-country analysis.

Ji et al. (2014) and Chung (2016) explored 73 and 58 recipient countries, respectively. Ji et al. (2014) utilized urbanization as a variable because urbanization has been identified as a key driving factor of economic growth. They conducted a panel data analysis using data from 1996 to 2012 development stages. Their findings revealed that the impact of ODA on economic growth varies depending on the development level in a recipient country. Chung (2016) investigated the impact of Korean ODA on the economic growth of the recipient countries between 1992 and 2014. The impact of ODA was statistically significant in the third period (2011–2014), representing that ODA’s effect between 2011 and 2014 was much higher compared to the other two periods (1992 to 2000 and 2001 to 2010).

Both Kim and Jang (2012) and Hwang et al. (2016) targeted African countries. Kim and Jang (2012) conducted an empirical study using data from 51 African countries. The data covered the periods between 1995 and 2010. Since the importance of ICT is rising rapidly, this study focused on ICT ODA capital inflow. A quadratic equation was developed to examine the diminishing marginal effect of capital inflow. The empirical results show that while in the short run, ICT ODA promotes economic growth, the growth impact of ICT ODA declines in the long run. Hwang et al. (2016) used pooled regression, panel analysis, and spatial panel analysis in conducting a comparative analysis. The empirical model consists of population, sanitation expenditure, per capita GDP, trade, ODA, and governance for explaining the rate of economic growth. Trade openness and ODA inflow were found to be consistently and statistically significant for all the estimations. This means that the ODA’s impact on economic growth rate is robust; it does not depend on the estimator.

Yoon (2018) collected data from 38 Korean ODA priority partner countries from 1996 to 2013. Deviating from the path taken by the other existing studies, this study attempted to investigate the time-lag effect of ODA on economic growth by dividing the recipients countries by region. In all the regions—Asia, Africa, and South America—the Korean ODA was found to have a significant time-lag, thus promoting economic growth in the recipient countries. Kang and Kim (2019) utilized the most extensive dataset consisting of 137 recipient countries and covering the period from 2002 to 2017. Utilizing the vector-autoregressive (VAR) model, they found that science and technology ODA has the most powerful promotion impact on recipient countries than any other ODA.

Unlike the literature above, Choi (2015) adopted a time-series analysis to investigate the relationship between ODA and economic growth in four South American countries: Brazil, Argentina, Venezuela, and Peru. This study employed a reduced-form equation consisting of ODA, FDI, and trade openness. While other studies have focused on ODA’s impact on the economic growth of recipient countries, this study wanted to compare the effectiveness of ODA and FDI under the control of trade openness. Its empirical results revealed that ODA and FDI have a distinct effect in each country. For example, FDI was found to have a positive impact in Brazil and Argentina, while ODA promoted the economic growth in Argentina, Venezuela, and Peru.

Several studies covering the relationship between foreign capital and economic growth have focused on specific regions. For example, Jayaraman and Lau (2009) and Kumi et al. (2017) covered Pacific island countries and the Sub-Saharan Africa region, respectively. On the other hand, Teboul and Moustier (2001) used ODA and FDI as proxies of foreign aid that may have a positive impact on economic growth, while Moreira (2005) adopted a panel approach to estimate the Harrod-Domar model, which is used to explore the impact of foreign aid on domestic income and was also utilized in Kim and Jang (2012). In line with Kim and Jang’s (2012) research, Moreira (2005) also found that the effect of foreign aid on economic growth diminishes in the long run. Karras (2006) also conducted an empirical research for 71 aid-receiving developing countries, covering the period from 1960 to 1997. He found evidence to the effect that ODA promotes economic growth in recipient countries. Tekin (2012) and Driffield and Jones (2013) focused on developing countries. The results of a panel bootstrap causality analysis in Tekin (2012) show no significant relation among foreign aid, trade openness, and economic growth for African LDCs. Driffield and Jones (2013) adopted a system approach for measuring the different impacts of ODA, FDI, and migrant remittances on economic growth. Their findings revealed that there is a positive relationship between overseas capital flow and the economic growth of developing countries.

### The relationship between economic growth and GHG emissions

The relationship between environmental degradation and economic growth has been an issue of debate in the environmental economics field. Kuznets (1955) proposed a concept that advocates the distribution of wealth when a country’s economy reaches a certain level. Grossman and Krueger (1992) borrowed Kuznets’ theory and applied it in environmental economics. The environmental Kuznets curve (EKC) explains the relationship between economic growth and environmental degradation using an inverted-U shape curve. Nowadays, with the increasing awareness about climate change, GHGs are considered as a proxy for environmental degradation. Global efforts against climate change are a replica of the EKC hypothesis because most of the Annex I parties in the Kyoto Protocol are OECD countries (Jin and Kim 2020).

There have been raging debates on the existence of EKC, with many studies arguing that EKC exists and others arguing that it does not exist. Harbaugh et al. (2002) emphasizes that although the EKC can exist, the confirmation of this hypothesis is so vulnerable because it highly depends on the sample size. The EKC hypothesis studies on 25 African countries, Tunisia, and 16 EU countries have failed to find evidence on its existence (Zoundi 2017; Jebli and Youssef 2015; Boluk and Mert 2014).

According to Bernard et al. (2015), the EKC hypothesis is generally confirmed in only developed countries, such as those in the OECD group, and not in developing countries. However, in both developed and developing countries, the results for the confirmation of the EKC hypothesis depend on the sample size and the estimation method. For instance, Özokcu and Özdemir (2017) found N-shaped EKC evidence for 26 OECD countries. Al-Mulali and Ozturk (2016) also revealed that 27 advanced countries exhibited the EKC process. On the other hand, Shafiei and Salim (2014) did not find even a single piece of evidence on the existence of the EKC in 29 OECD countries. Likewise, Boluk and Mert (2014) also reported that the EKC did not exist in 16 EU countries. There are other literatures insisting that the evidence of EKC hypothesis can partially exist.

Işik et al. (2019a, b) attempted to describe the EKC relationship for the states level in the USA. Both research conducted panel data analysis. Işik et al. (2019a) and Işik et al. (2019b) analyzed for 50 states and 10 states in the USA, respectively. The evidence of EKC hypothesis was found for 14 out of 50 states and five out of 10 states with renewable and fossil energy consumption as control variable; Jin and Kim (2020) also found the partial evidence of EKC hypothesis for Annex I countries. Only five out of 34 countries have confirmed EKC relationship.

Işik et al. (2017a, b) adopted time-series analysis to investigate the relationship between economic growth and CO_2_ emissions in Greece. Autoregressive distributed lag (ARDL) approach and Granger causality were used. They revealed that economic growth, financial development, international trade, tourism and CO_2_ emissions have long-run equilibrium relationship. Even though they did not use EKC framework, in Greece, economic growth has increased CO_2_ emissions under control of the other variables: financial development, international trade, tourism. Ongan et al. (2020) also used time-series analysis for confirming EKC in the USA. The decomposition for income series was conducted to describe the exact relationship between income and environmental degradation. The analysis framework is same with Işik et al. (2017a, b): ARDL approach and Granger causality analysis. The empirical results show that under income series decomposition, they found the evidence of EKC hypothesis in the USA, while undecomposed model shows no relationship.

For the panel analysis, the developing countries targeted were ASEAN-4 and 25 African countries (Liu et al. 2017; Zoundi 2017). Dogan et al. (2020) conducted EKC research focusing on BRICST countries, although all of BRICST countries are not developing countries. No evidence on the EKC hypothesis was found for all three papers. The EKC study on developing countries suffers from a relative absence of research. This is attributed to the unavailability of data in developing countries. The assertion of Bernard et al. (2015) can be a reliable hypothesis when it comes to the similarity between the international movement for climate mitigation and the EKC hypothesis (Jin and Kim 2020). Therefore, the confirmation of EKC hypothesis in developing countries, which are the recipient countries of Korea in this study, can also be a meaningful attempt.

The existing literature, in the “The impact of ODA on the economic growth of recipient countries” section, has analyzed the effectiveness of ODA on the economic growth of recipient countries using various determinants. However, this literature uses various models, such as the Harrod-Domar model and the growth model of Solow, as a theoretical basis. Previous studies, as outlined in the “The relationship between economic growth and GHG emissions” section, have also attempted to investigate the relationship between economic growth and GHG emissions. While each study has its own contribution to the existing literature, the studies are limited by the fact that they only examined the relationship between foreign capital inflow and economic growth, ignoring the crucial issue of climate change. On the other hand, as an indirect model, this study adopts a simultaneous equation approach based on the Cobb-Douglas production function to consider the domestic factors of production. Bae and Kim (2012) suggest that the most appropriate way to examine the impact of factors of production on GHGs emissions is using two equational frameworks.

Based on Bae and Kim’s (2012) approach, we designed the following research steps for an indirect estimation of the ODA’s impact on CO_2_ emission. After confirming the impact of ODA on economic growth, we attempted to investigate its effect on CO_2_ emissions by examining the relationship between CO_2_ emissions and economic growth. This study employed the modified impact, population, affluence, and technology (IPAT) model, which is effective in establishing the impact of ODA, compared to the national GDP and other control variables for measuring the direct impact of ODA on carbon emissions.

## Data and the empirical model and methods

### Data

The purpose of this study is to measure the impact of ODA on CO_2_ emissions for the recipient countries of Korea. For the empirical analysis, the annual data of 30 recipient countries^2^ of Korea were collected. The data collected covered the period from 1993 to 2017. The variables adopted in this study consist of the factors of production, population, ODA, economic growth, and CO_2_ emissions. Table 1 presents the descriptive statistics of each variable. We obtained the annual panel dataset for per capita GDP in constant 2010 US$ as a proxy of economic growth (World Bank 2019). In this study, the factors of production, based on the economic theory, consist of energy consumption, capital, and labor variables. Energy consumption data, which are represented by the total primary energy supply (TPES) in ktoe, and the population in millions are derived from World Energy Balances (IEA 2019a) and World Indicators (IEA 2019b), respectively. The gross fixed capital formation, as a proxy of capital and the net ODA received in millions constant 2010 US$, and labor force data in millions were obtained from same source as that of economic growth (World Bank 2019). Lastly, CO_2_ emissions data in Mt were derived from the European Commission (2019).

**Table 1:** Descriptive statistics

Variable	Mean	SD	Max	Min	Obs.
CO_2_ emissions (*CO*_2_)	52.21	77.74	531.97	0.53	*N* = 30, *T* = 25, *N* × *T* = 750
Economic growth (*Y*)	2148.31	1550.68	7626.00	207.01	
Energy consumption (*E*)	27,084.85	38,271.07	244,066.00	209.07	
Capital (*K*)	26,970.84	85,754.99	1,149,118.36	56.22	
Labor (*L*)	18.44	22.53	129.62	1.00	
ODA (*O*)	944.21	953.49	11,331.36	10.83	
Population (*P*)	46.46	54.58	264.65	4.20	

As described above, we developed two estimation models for measuring the ODA’s effectiveness on CO_2_ emissions—the direct and indirect models. We adopted different theoretical frameworks for each model so that the each model could have a constructed hypothesis that penetrates the model as follows. For the direct model, under the control of economic activity and population, the ODA would have a direct impact on CO_2_ emissions. On the other hand, the indirect model focused on measuring the indirect effect of economic growth on CO_2_ emissions using an indirect pathway. That is, the indirect model aimed at examining the impact of ODA on CO_2_ emissions via economic growth.

### Direct model

The direct model is based on the IPAT model. The IPAT model aims at identifying the determinants of the environmental impact by population, affluence, and technology variables, and it has a form of decomposition equation suggested by Ehrlich and Holdren (1971) (Eq. (1)).

1 $$ I\left(\mathrm{environmental}\ \mathrm{impact}\right)=P\left(\mathrm{population}\right)\times A\left(\mathrm{affluence}\right)\times T\left(\mathrm{technology}\right) $$

For this model, the per capita GDP and energy intensity are used as the indicators of affluence and technology, respectively. However, York et al. (2003) outlines the defects of the IPAT model. When part of the equation is pre-determined, the rest the equation is determined automatically. This problem is induced because the IPAT model adopts a deterministic measure method. Therefore, the way to estimate the IPAT model using a stochastic approach (stochastic impacts by regression on population, affluence, and technology, STIRPAT) was proposed (Dietz and Rosa 1997) (Eq. (2)).

2 $$ {I}_i=a{P}_i^b{A}_i^c{T}_i^d{e}_i $$

Alphabets from *a* to *d* indicate the coefficients’ corresponding explanatory variables. *e*_*i*_ denotes the stochastic error. The STIRPAT model allows the IPAT model to be estimated stochastically so that the effectiveness of each variable on the environmental impact can be measured. In this study, we modified this model by substituting technology with foreign aid (ODA). Therefore, the model estimated in this study can be described as Eq. (3). This equation measures the impact of the ODA size compared to the GDP on the economic growth of a recipient country, under the consideration of economic growth and population effect. We estimated Eq. (3) linear regression using the natural logarithm transformation, as shown in Eq. (4).

3 $$ {CO}_{2, it}=a\times {P}_{it}^b\times {A}_{it}^c\times {T}_{it}^d\times {e}_{it} $$

4 $$ \ln {CO}_{2, it}=a+b\ln {P}_{it}+c\ln {\left(\frac{GDP}{P}\right)}_{it}+d\ln {\left(\frac{ODA}{GDP}\right)}_{it}+{e}_{it} $$

where *i* and *t* each indicate the cross section and time-series in panel analysis, respectively. *e*_*it*_ is identically and independently distributed error term.

### Indirect model

As mentioned above, we used the indirect model to show the impact of ODA on CO_2_ emissions in recipient countries via the economic growth pathway, based on the simultaneous equation system. This was achieved through the following two steps: first, we estimated the relationship between ODA and economic growth using the Cobb-Douglas production function. Energy, capital, and labor were well-used factors of production in existing literature. Energy can be a critical factor for economic growth, as existing literature includes energy as a factor of production (Işik et al. 2017a, b; Işik and Radulescu 2017).

Besides, foreign capital inflows, such as ODA, can be substitutable factors of production according to Nam and Kim (2018). Lee (2014) also points out that foreign aid can be more efficient when ODA is combined with factors of production such as the capital and labor of the recipient countries. Therefore, in this context, we used four factors of production, as presented in Eqs. (5) and (6).

5 $$ Y=f\left(E,K,L,O\right) $$

6 $$ \ln {Y}_{it}={\beta}_0+{\beta}_1\ln {E}_{it}+{\beta}_2\ln {K}_{it}+{\beta}_3\ln {L}_{it}+{\beta}_4\ln {O}_{it}+{\varepsilon}_{it} $$

where *Y*, *E*, and *O* denote per capita GDP, per capita TPES, and the net ODA received, respectively. The mean of the subscripts is same as that in Eq. (4). Measuring the impact of ODA on economic growth by estimating Eq. (6), we proceeded to estimate the next equation, which is based on the EKC hypothesis, as follows:

7 $$ \ln {CO}_{2, it}={\alpha}_0+{\alpha}_1\ln {Y}_{it}+{\alpha}_2{\left(\ln {Y}_{it}\right)}^2+{\epsilon}_{it} $$

The aim of Eq. (7) was to investigate the relationship between economic growth and CO_2_ emissions. According to Jin and Kim (2020), researchers have tested different exogenous variables for testing the EKC hypothesis. However, the exogenous variables must be the ones that directly affect CO_2_ emissions with weak or no endogeneity. We decided not to include the additive variables because variables such as trade are not suitable for developing countries. Through Eq. (6), we estimated the impact of ODA on economic growth. We also estimated the CO_2_ emissions on economic growth using Eq. (7). Since the estimated coefficients in Eqs. (6) and (7) are the elasticity, the indirect impact of ODA on CO_2_ emissions via economic growth can be calculated as follows:

8 $$ \frac{\partial \ln {CO}_{2, it}}{\partial \ln {O}_{it}}=\frac{\partial \ln {CO}_{2, it}}{\partial \ln {Y}_{it}}\times \frac{\partial \ln {Y}_{it}}{\partial \ln {O}_{it}}=\left({\alpha}_1+2{\alpha}_2\overline{\ln {Y}_{it}}\right)\times {\beta}_4 $$

### Estimation methods

We used panel data analysis to take account of the heterogeneity of each country. The panel estimators, fixed and random effect estimators, can be used to represent the different situation of each country in this model. The different situation is called the unobserved heterogeneity. The basic panel linear regression model is as follows:

9 $$ {y}_{it}=\alpha +\beta {x}_{it}+{u}_i+{e}_{it} $$

where, *y* and *x* denote the dependent variable and regressors, respectively. *α* denotes the common intercept and *β* is the estimated coefficient of the corresponding regressor, which is a homogeneous slope. *u*_*i*_ represents the unobserved heterogeneity. When it comes to estimating the unobserved heterogeneity, the panel linear model can be classified into two: the fixed effect and random effect models. The fixed effect and random effect models consider the unobserved heterogeneity parameter to be estimated and the random variable, respectively.

The consistency condition can be satisfied when OLS estimators are derived for Eq. (9) without a relationship between explanatory variables and residuals. This is described as the random effect hypothesis. Since the first-order autocorrelation problem can be induced when the equation is estimated by OLS under the satisfaction of the random effect hypothesis condition, the random effect model should be estimated by generalized least squares. However, the random effect hypothesis is too strict to be satisfied. Therefore, most panel linear regressions adopt the fixed effect model that assumes the unobserved heterogeneity as the invariant estimation. For a more efficient estimator, we can use the Hausman test that verifies the covariance between regressors and unobserved errors (Hausman 1978). If the covariance between the explanatory variables and the unobserved errors is not zero and statistically significant, the fixed effect model is more efficient than the random effect model.

## Empirical results

### Direct model results

Table 2 presents the direct model results using the fixed effect model. For comparison purposes, we present the fixed and random effect estimators with Hausman results. According to the results of the Hausman test, test statistics rejects the null hypothesis at the 1% significance level, which means that the fixed effect estimator is more efficient than the random effect model. The adjusted *R*-squared value indicates that the fixed effect model has high explanatory power that is enough to explain the model. For the estimated coefficients, there is no huge scale-gap between the fixed and random effect models. It seems that the estimated parameters are robust; they do not depend on the estimation method.

**Table 2:** Linear regression results of the direct model

Variable	Fixed effect	Random effect
	Coefficient (std. err)	Coefficient (std. err)
Intercept	− 22.2465 (0.9813) ***	− 20.6678 (0.8660) ***
Population	1.2906 (0.0696) ***	1.1642 (0.0595) ***
Per capita GDP	0.4096 (0.0464) ***	0.4896 (0.0419) ***
ODA per GDP	− 0.0422 (0.0126) ***	− 0.0395 (0.0125) ***
Adjusted R-squared	0.9826	0.6734
Hausman test statistics H_0_: Random effect is more efficient		21.8168 ^c^

***Rejection of the null hypothesis at the 1% significance level

All the estimated coefficients are statistically significant at the 1% significance level, thus affecting CO_2_ emissions. The population and per capita GDP, as proxies of economic growth, are derived as the increasing factors of CO_2_ emissions. Since the regressors in the equation are a form of natural logarithm, the estimated coefficients denote elasticity, representing the growth rate of the corresponding explanatory variable compared to the growth rate of dependent variable. According to the fixed effect results, a 1% increase in population leads to about 1.29% increase of CO_2_ emissions. Compared to the elasticity of the population, per capita GDP has a small impact on CO_2_ emissions. A 1% increase in per capita GDP induces an increment of about 0.41% of CO_2_ emissions. We derived that the main explanatory variable, ODA per GDP, has a mitigation effect. When ODA per GDP increases by 1%, CO_2_ emissions are reduced by about 0.04%

For the diagnostics, we conducted a residual normality test and cross-sectional dependence. Table 3 shows the results. The diagnostic test results suggest strong evidence that at the 1% significance level for both diagnostics, the residual does not follow the normal distribution and cross-section dependence. This means that the results of the fixed effect estimator in Table 2 can be biased. Therefore, we decided to use the mean group (MG) estimator method, including MG and an augmented MG (AMG) estimator, to estimate a direct model that is robust to cross-section dependence.

**Table 3:** Diagnostic test results for the fixed effect of the direct model

Test	Test statistics (*P* value)
Normality test (Jarque–Bera) H_0_: Random effect is more efficient	55.1167 (0.0000)***
Cross-section dependence test H_0_: No cross-section dependence exists	BP LM: 3242.071 (0.0000)*** Pesaran LM: 95.1686 (0.0000)***

***Rejection of the null hypothesis at the 1% significance level

Table 4 shows the estimation results of the MG estimator. For both the MG and AMG estimators, the ODA per GDP has a statistically significant impact on CO_2_ emissions by a similar scale of impact with the fixed effect model. According to the MG estimator results, a 1% increase in ODA per GDP results in a 0.04% decrease of CO_2_ emissions. While the impact of the population on CO_2_ emissions is smaller than the fixed effect estimator, it is still bigger than per capita GDP. Overall, for the recipient countries of Korea, population, and economic growth have increased CO_2_ emissions, and the population has a bigger impact than economic growth. By deriving the elasticity of ODA per GDP on CO_2_ emissions, the size of ODA compared to economic growth can directly affect CO_2_ mitigation.

**Table 4:** The estimated MG estimators of the direct model

Variable	MG estimator	AMG estimator
	Coefficient (std. err)	Coefficient (std. err)
Intercept	− 21.2037 (3.6932) ***	− 15.3825 (8.2616) ***
Population	0.9791 (0.2287) ***	0.7001 (0.4496)
Per capita GDP	0.5746 (0.2384) **	0.4880 (0.2600) *
ODA per GDP	− 0.0392 (0.0128) ***	− 0.0380 (0.0130) ***
RMSE	0.0791	0.0739

RMSE means root mean squared error

*, **, and ***Rejection of the null hypothesis at the 10%, 5% and 1% significance level, respectively

### Indirect model results

To measure the indirect impact of ODA on CO_2_ emissions via economic growth, we estimate the Cobb-Douglas production function presented by Eq. (6). Production function estimation with panel data involves the scale effect of each country. Furthermore, the EKC hypothesis is based on per capita variables, which are used to investigate the relationship between per capita income and environmental degradation. Therefore, we decide to estimate the Cobb-Douglas production function using the per capita form of all variables. In line with the direct model estimation, we start from the fixed and random effect estimation.

Table 5 presents the linear regression results of the indirect model. The Hausman test results indicate that the fixed effect model is more efficient, and the adjusted *R*-squared value reveals that the fixed effect model has more explanatory power than the random effect model, as observed in the direct model results. All the test statistics reject the null hypothesis of no impact on the dependent variable at the 1% significance level. The traditional factors of production, capital and labor force, have a significant impact on economic boost. Energy consumption also functions as the driving force of economic growth. In short, based on the fixed effect results, a 1% increase in energy consumption, gross fixed capital formation and labor force increase per capita GDP by 0.20%, 0.25%, and 1.45%, respectively. On the other hand, the foreign capital inflow through ODA affects economic growth negatively. When there is a 1% of the net ODA received, the economic growth retreats.

**Table 5:** The results of production function estimation in the indirect model

Variable	Fixed effect	Random effect
	Coefficient (std. err)	Coefficient (std. err)
Intercept	6.0853 (0.1908) ***	4.7739 (0.2730) ***
Energy consumption	0.2010 (0.0418) ***	0.3061 (0.0300) ***
Capital	0.2486 (0.0277) ***	0.2814 (0.0395) ***
Labor force	1.4456 (0.1198) ***	0.9164 (0.1523) ***
Net ODA received	− 0.0165 (0.0053) ***	− 0.0233 (0.0065) ***
Adjusted *R*-squared	0.9662	0.6189
Hausman test statistics H_0_: Random effect is more efficient		151.9660 ***

***Rejection of the null hypothesis at the 1% significance level

The interesting point of the production function estimation is that the impact of the labor force on economic growth is much higher than capital, which is consistent with the results of Jin and Kim (2018b). For developed countries, such as the member countries of OECD, the impact of capital is much higher than labor force because the social and economic infrastructure is already set and is ready for economic development. However, developing countries require the setting up of infrastructure for economic development before capital investment. This is the reason as to the why the labor force has a high impact on economic growth in developing countries.

To follow the same analysis framework with the direct model, we conduct a diagnostic test for the production function estimation. Likewise, the normality test rejects the null hypothesis of normality at the 1% significance level. The cross-section dependence test results indicate the presence of dependency. Given the non-normality and cross-sectional dependence, the estimator can be biased. In this case, we adopt MG estimators in estimating the elasticities of each factor of production (Table 6).

**Table 6:** Diagnostic test results for the fixed effect of the direct model

Test	Test statistics (*P* value)
Normality test (Jarque-Bera) H_0_: Random effect is more efficient	1891.955 (0.0000) ***
Cross-section dependence test H_0_: No cross-section dependence exists	BP LM: 4102.232 (0.0000) *** Pesaran LM: 124.3308 (0.0000) ***

***Rejection of the null hypothesis at the 1% significance level

Table 7 shows the results of the MG estimator. For both estimators, except the ODA variable, all the estimated coefficients are statistically significant at the 1% significance level. However, in contrast with the direct model results, the estimated coefficients of the MG estimator are different from those of the fixed effect estimator. To explain the Cobb-Douglas production function based on RMSE, we choose to use the AMG estimator. The empirical results propose that while energy consumption, capital, and labor force promote economic growth, ODA has a negative impact on economic growth. A 1% increase in energy consumption, capital, and labor force induces an economic growth of 0.27%, 0.13%, and 0.56%, respectively. On the other hand, for the AMG estimator, an ODA increase by 1% decreases the economic growth by 0.02% with a 10% significance level. Similar to Griffin and Enos’s (1970) report, our study derives that ODA hinders the economic growth of the recipient countries.

**Table 7:** The results of the MG estimators in the indirect model

Variable	MG estimator	AMG estimator
	Coefficient (std. err)	Coefficient (std. err)
Intercept	4.4770 (0.5950) ***	5.3651 (0.3331) ***
Energy consumption	0.3765 (0.0695) ***	0.2676 (0.0461) ***
Capital	0.2698 (0.0344) ***	0.1317 (0.0281) ***
Labor force	0.6905 (0.2186) ***	0.5577 (0.2831) **
Net ODA received	0.0014 (0.0089)	− 0.0164 (0.0086) *
RMSE	0.0481	0.0347

RMSE means root mean squared error

*, **, and ***Rejection of the null hypothesis at the 10%, 5%, and 1% significance level, respectively

The EKC hypothesis is tested and the impact of economic growth on CO_2_ emissions is measured by Eq. (7). To estimate the EKC equation, we adopt the long-run estimator represented by a fully modified OLS (FMOLS) and a dynamic OLS (DOLS) suggested by Pedroni (2000) and Mark and Sul (2003), respectively. These methods can correct the biases and capture the long-run dynamics under the consideration of cross-section dependence.

The estimation results of Eq. (7) using FMOLS and DOLS are shown in Table 8. For both estimators, we find the empirical evidence supporting the EKC hypothesis. Since the EKC equation is in a quadratic form, the combination of the positive and negative coefficients corresponding economic growth and the EKC variable shows the evidence of an EKC curve. The turning points of the curve can be calculated by $$ \exp \left(-\frac{\alpha_1}{2{\alpha}_2}\right) $$. The calculated turning points are about 9236 and 5241 as of per capita GDP in 2010 US$.

**Table 8:** The estimated coefficients of the EKC equation in the indirect model

Variable	FMOLS	DOLS
	Coefficient (std. err)	Coefficient (std. err)
Economic growth	2.8598 (0.1478) ***	2.7080 (0.5746) ***
EKC variable	− 0.1566 (0.0100) ***	− 0.1581 (0.0376) ***
Adjusted R-squared	0.9754	0.9926
Turning point	9236.39	5240.65
Calculated indirect elasticity of ODA on CO_2_ emissions	− 0.009	− 0.006

EKC variable indicates the quadratic form of economic growth

***Rejection of the null hypothesis at the 1% significance level

The last row of Table 8 shows the calculated indirect elasticity of ODA on CO_2_ emissions via economic growth. If there is a 1% increase of per capita ODA, a 0.006 to 0.009% reduction of CO_2_ is experienced. In other words, the indirect pathway, which is the impact of the economic growth from ODA on CO_2_ emissions, is limited. Compared to the descriptive statistics in Table 1, most of the recipient countries have not yet reached a turning point. This implies that the indirect effect of ODA on CO_2_ emissions can be expanded after the economic situation of the countries gets better.

## Conclusion

The purpose of the study was to measure the impact of ODA on CO_2_ emissions for the recipient countries of Korea. To accomplish this, we collected a panel dataset consisting of 30 countries for the period from 1993 to 2017. Considering the link between foreign capital inflow and economy and economy and environment, we divided our investigation into two models: the direct and indirect models. The empirical results can be summarized as follows. For the direct model, while the population and economic growth accelerate CO_2_ emissions, the ODA per GDP has a mitigation impact on CO_2_ emissions. In addition, the estimation results of the indirect model reveal that the factors of production, energy consumption, capital, and labor force have a positive impact based on the Cobb-Douglas production function assumption. Otherwise, the ODA affects economic growth negatively. The EKC hypothesis is empirically proven by estimators that can correct a serial correlation. Despite the combined results of the production function with the EKC equation, the long-run impact of ODA on CO_2_ emissions is derived as a weak mitigation.

The core results suggest some implications. The direct mitigation impact of ODA per GDP and indirect ODA’s weak mitigation impact indicate that ODA for the recipient countries should take economic situation of them into account to accomplish both climate mitigation for global perspectives and economic assistance of recipient countries. Even though we derive the empirical results that ODA has negative impact on economic growth based on production function, the robustness of impact and statistical significance are weak. Furthermore, we reveal the evidence of EKC hypothesis for the recipient countries, which provides the information that economic growth in developing countries can contribute to climate mitigation. Accordingly, the foreign capital inflow program to accelerate the economic growth of developing countries should be enhanced.

This study contributes to the existing literature in several ways. We estimate the direct and indirect impact of ODA on CO_2_ emissions. To measure the direct impact of ODA, we modify the IPAT model, which is frequently used to investigate the determinants of environmental degradation. Specifically for the indirect model, we refer to the existing literature so that we can construct a model investigating a pathway that is different from the ODA to CO_2_ emissions. The scale of impact is small, being derived as 0.006 to 0.009% of the mitigation impact by 1% increase of ODA. However, the estimated economic growth’s elasticity of CO_2_ emissions in the EKC equation suggests that the economic growth will eventually mitigate emissions. Based on these results, this study suggests that there is a future potential that ODA’s mitigation impact will increase by far when the economic growth of the recipient countries reaches a certain level.

Given the fact that global cooperation for climate change mitigation has been emphasized, the strategy for foreign capital inflow from advanced to developing countries should be on aware of this issue. Since the establishment of OECD DAC in 1960, numerous bilateral assistances focusing on the economic development of the recipient countries have been conducted. However, the empirical data used in this study starts from 1993, thus reflecting a relative recent trend. Based on the empirical results of this study, we suggest that to ensure sustainable development, future ODA take CO_2_ mitigation into account. Furthermore, the international aid and trade aimed at fostering the economic growth of developing countries should be expanded, even though the empirical results show that ODA has an insignificant impact on economic growth. Regardless of the impact of ODA on economic growth, when the economic situation and environmental policy in developing countries are improved and well-established, the effectiveness of climate change mitigation in the view of international cooperation will be accelerated.

We recognize the limitations of this study in following points. Firstly, we adopt the ODA as a proxy of capital inflow to developing countries. In real, various ways have been utilized to provide financial and technological aid. Secondly, we utilize the panel data analysis assuming homogeneous slope for each individual cross-section. In recent as we mentioned above, the existing literature covering the EKC hypothesis adopt the estimation technique that can handle heterogeneous slope. Future study can be pursued by as follows. We can extend the subject countries consisting of entire recipient countries. The different impact of ODA on GHG emissions according to income-level can be an interesting topic.

## Footnotes

1 https://unfccc.int/process-and-meetings/the-paris-agreement/what-is-the-paris-agreement

2 Thirty recipient countries of Korea include Algeria, Azerbaijan, Bangladesh, Bolivia, Cameroon, Colombia, Congo DR, Ecuador, Egypt, El Salvador, Guatemala, Indonesia, Jordan, Kenya, Kyrgyzstan, Morocco, Mozambique, Nigeria, Pakistan, Paraguay, Peru, Philippines, Rwanda, Senegal, Sri Lanka, Tanzania, Tunisia, Uganda, Uzbekistan, Vietnam.
